# Hybrid Low-Order and Higher-Order Graph Convolutional Networks

**DOI:** 10.1155/2020/3283890

**Published:** 2020-06-23

**Authors:** Fangyuan Lei, Xun Liu, Qingyun Dai, Bingo Wing-Kuen Ling, Huimin Zhao, Yan Liu

**Affiliations:** ^1^Guangdong Province Key Laboratory of Intellectual Property and Big Data, Guangzhou 510665, China; ^2^School of Electronic and Information, Guangdong Polytechnic Normal University, Guangdong, Guangzhou 510665, China; ^3^School of Information Engineering, Guangdong University of Technology, Guangdong, Guangzhou, China; ^4^School of Computer Sciences, Guangdong Polytechnic Normal University, Guangdong, Guangzhou 510665, China

## Abstract

With the higher-order neighborhood information of a graph network, the accuracy of graph representation learning classification can be significantly improved. However, the current higher-order graph convolutional networks have a large number of parameters and high computational complexity. Therefore, we propose a hybrid lower-order and higher-order graph convolutional network (HLHG) learning model, which uses a weight sharing mechanism to reduce the number of network parameters. To reduce the computational complexity, we propose a novel information fusion pooling layer to combine the high-order and low-order neighborhood matrix information. We theoretically compare the computational complexity and the number of parameters of the proposed model with those of the other state-of-the-art models. Experimentally, we verify the proposed model on large-scale text network datasets using supervised learning and on citation network datasets using semisupervised learning. The experimental results show that the proposed model achieves higher classification accuracy with a small set of trainable weight parameters.

## 1. Introduction

Convolutional neural networks (CNNs) have achieved great success in grid structured data such as images and videos [1, 2]. It is attributed to a series of filters of convolutional layers from the CNNs that can obtain local invariant features. Compared to a regularized network, the number of neighbors of a node in a graph network may be different. Therefore, it is difficult to directly implement the filter operator in an irregular network structure [3].

In the graph network, the nodes and the connecting edges between them contain abundant network characteristic information. A graph convolutional network (GCN) aggregates the neighborhood nodes to realize continuous information transmission based on a graph network. By making full use of this information, a GCN can effectively achieve tasks such as classification, prediction, and recommendation.

A graph convolutional network (GCN) generalizes traditional convolutional neural networks (CNNs) to the graph domain. The GCN methods are mainly divided into two categories [3], the frequency domain-based methods [4–6] and the spatial domain-based methods [7, 8].

In the spatial domain, to simulate the convolution operation of the traditional CNN on an image, the convolution operation aggregates the information of the neighborhood nodes [7–10]. Henaff et al. [11] proposed a smoothed parametric spectral filter to realize localization and to preserve the parameters of filters independent of the input dimension. One of the key challenges is that the number of neighborhood nodes in the network irregularly changes.

In the frequency domain, Bruna et al. [5] were the first ones to extend CNN-type architectures to graphs. Cao et al. [12] applied a generalized convolutional network to the graph frequency domain using the Fourier transform. In this method, eigenvalue decomposition is performed on the neighborhood matrix. To reduce the computational complexity, Defferrard et al. [13] proposed the Chebyshev polynomial of the eigenvalues of the graph Laplacian to achieve efficient and localized graph convolutional operation filters. Kipf and Welling [6] proposed a classical GCN, which was approximated by a first-order Chebyshev polynomial. This approach reduces the computational complexity but introduces truncation errors. This introduction results in the inability to capture high-level interaction information between the nodes in the graph, and it also limits the capabilities of the model. The information propagation process in the graph is related not only to its first-order neighborhood but also to its higher-order neighborhood.

Abu-El-Haija et al. [14, 15] proposed the high-order convolutional network layer on a graph that used linear combination of the high-order neighborhood basis of the GCN [6]. Tiao et al. [16] proposed a Bayesian estimation approach via the stochastic variational inference in the adjacency matrix of the graph. Levie et al. [17] proposed Cayley polynomials to compute the localized regular filters of the interest frequency bands of graphs. Therefore, the rational use of second-order neighborhoods, third-order neighborhoods, and other high-order neighborhood information will be beneficial to classification prediction accuracy [14–16, 18–20].

**Figure 1:**
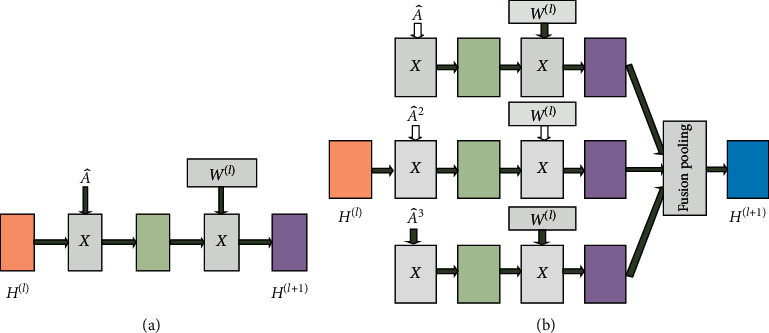
The graph convolutional layer of our model. (a) First-order graph convolutional layer of the Kipf and Welling [6] model. The input is *H*^(*l* − 1)^, the output is *H*^(*l*)^, and the trainable parameter is *W*^(*l*)^. (b) The 3rd order graph convolutional layer of our HLHG model. Different order neighborhood matrices share the trainable weight.

Based on the classical GCN [6], to make full use of the high-order and low-order neighborhood information, we propose a novel hybrid low-order and higher-order graph convolutional network (HLHG). As shown in Figure 1, the graph convolutional layer of our model is simple and effective at capturing the high-order neighborhood information, nonlinearly combining the different order neighborhood information. The contributions are summarized as follows:

1. We propose a new fusion pooling layer to achieve high-order neighborhood fusion with the low-order neighborhood of graph networks
2. We propose a low-order neighborhood and high-order neighborhood weight sharing mechanism to reduce the computational complexity and number of parameters of the model
3. The experimental results show that our HLHG achieves state-of-the-art performance in both the text network classification with supervised learning and the citation network with semisupervised learning

The rest of the paper is organized as follows. In Section 2, the related theoretical basis such as the graph convolution and the high-order graph convolution are introduced. In Section 3, the general information fusion pooling for the high-order neighborhood is presented. Then, the proposed model and its variant are presented. The computational complexity and parameter quantity of the proposed model are also theoretically analyzed. In Section 4, our proposed model is verified and the corresponding analysis are presented. Finally, Section 5 concludes the paper.

## 2. Related Theoretical Background

In this section, the related theoretical basis will be introduced, including the graph convolutional network (GCN).

### 2.1. Graph

Given a graph *G*, its nodes set *V*, and its edges *E*, the graph is represented as *G*=(*V*, *E*). If nodes *V*_*i*_ and *V*_*j*_ are connected, then *E*_*ij*_=1; otherwise, *E*_*ij*_=0. The information in the graph propagates along with the edge *E*. It also applies when considering the network node self-loop, which means that *E*_*ii*_=1. Assuming that the information that is propagated by each node in the graph network is *x* ∈ *R*^*r*^, the information matrix in the graph is *X* ∈ *R*^*n*×*r*^, where *n* is the total number of nodes in the graph network and *r* is the dimension of the information feature. It assumes that if the loop graph network *G* is represented as $\tilde{G}$, then the adjacency matrix of the graph network $\tilde{G}$ is represented as $\tilde{A}=\left(A+I\right)$. The degree matrix of $\tilde{A}$ in the graph network $\tilde{G}$ is the diagonal matrix, ${\tilde{D}}_{ii}=\sum_{j}{\tilde{A}}_{ij}$.

### 2.2. Graph Convolutional Network

In the given graph *G*, there are two signals *f*=(*f*_1_,…,*f*_*n*_)^*T*^ and *g*=(*g*_1_,…,*g*_*n*_)^*T*^. The graph's Fourier transforms are defined as $\hat{f}=\Phi^{T}f$and $\hat{g}=\Phi^{T}g$, where Φ is the orthonormal eigenvalues of the graph Laplacian of graph *G*. The same as in Euclidean space, the spectral graph convolution operation of *f* and *g* is given as an elementwise product as follows:

(1) $$\begin{array}{c} g*f=\Phi \left(\left(\Phi^{T}g\right)\circ \left(\Phi^{T}f\right)\right)=\Phi \hat{G}\Phi^{T}f, \end{array}$$

where $\hat{G}=\text{diag}\left({\hat{g}}_{1},\ldots ,{\hat{g}}_{n}\right)\;$represents the diagonal matrix of $\hat{g}$.

Defferrard et al. [13] utilized the *k*-th order polynomial filters based on Chebyshev to represent the graph convolutional operation of Laplacian $\hat{G}=\sum_{i}\alpha_{i}\Lambda^{i}$, where *α*_*i*_ denotes the coefficients andΛ represents the eigenvalues of the Laplacian.

Kipf and Welling [6] propose the classical graph convolutional neural network model based on the Fourier transform, $g*f=\alpha \tilde{A}f$. The GCN model approximates the model using a first-order Chebyshev polynomial. The propagation model in the graph network is as follows:

(2) $$\begin{array}{c} H^{\left(l+1\right)}=\sigma \left({\tilde{D}}^{-\left(1/2\right)}\tilde{A}{\tilde{D}}^{-\left(1/2\right)}H^{\left(l\right)}W^{\left(l\right)}\right), \end{array}$$

where *H*^(*l*)^ denotes the information propagation matrix; *W*^(*l*)^ represents the trainable weight of layer *l*; when *l*=0, *H*^(0)^=*X* ∈ *R*^*n*×*r*^, which represents the initial input value of the GCN; *σ*(.) denotes the activation function. To reduce the computational complexity, the convolution operator in the graph is defined by a simple neighborhood average. However, the convolutional filters are too simple to capture the high-level interaction information between the nodes in the graph. Therefore, the classification accuracy on citation network datasets is low.

Abu-El-Haija et al. [14, 15] propose a high-order graph convolutional layer model based on the GCN for semisupervised node classification. The propagation model of the high-order graph convolution is as shown in formula (3). In this model, the transfer function of the (*l*+1)-th layer is a column concatenation from the first order to the *p* order in the *l*-th layer, which is the linear combination of the high-order neighborhood. In the propagation model, the different order neighborhoods of the same layer use different weight parameters:

(3) $$\begin{array}{c} H^{\left(l+1\right)}=\sigma \left(\left.B^{\left(0\right)}H^{\left(l\right)}W_{0}^{\left(l\right)}\right|\ldots \left|B^{\left(p\right)}H^{\left(l\right)}W_{p}^{\left(l\right)}\right.\right), \end{array}$$

where $B={\tilde{D}}^{-\left(1/2\right)}\tilde{A}{\tilde{D}}^{-\left(1/2\right)}$. However, as the network layers deepen, the dimensions of *H*^(*l*+1)^ will increase and propagate between layers. Therefore, the number of trainable weight parameters will be more, and the training resource will also be increased to learn the optimized dimension of the weight.

## 3. Method

When the message passes through the graph network, the nodes will receive latent representations from their first-hop nodes and from their N-hop neighbors every time. In this section, we propose a model to nonlinearly aggregate the trainable parameters, which can choose how to mix latent messages from various hop nodes.

### 3.1. General Information Fusion Pooling

The information propagation of the graph network is passed along the edges between the vertices in the graph. It assumes that the graph network *G*=(*V*, *E*) is an undirected graph. The general procedure of fusion pooling is described as follows. It assumes that the *k*-th order neighborhood matrix is *A*^(*k*)^=[*a*_*ij*_^(*k*)^], and the result after the fusion pooling operator is Pmax(*A*^(0)^,…,  *A*^(*k*)^)=*Z*^(*k*)^=[*z*_*ij*_^(*k*)^], where *z*_*ij*_^(*k*)^=max(*a*_*ij*_^(1)^, *a*_*ij*_^(2)^,…, *a*_*ij*_^(*k*)^)) and *k* represents the hop from the given node.

Here, is an example to show how to fuse the different order neighborhoods. For a given adjacency matrix $\hat{A}$, assume that *h*_1_ denotes the first-order neighborhood and *h*_2_ denotes the second-order neighborhood.

If $h_{1}=\hat{A}XW_{1}=\left[\begin{array}{cc} 1 & 0\\ 1 & 1 \end{array}\right]$ and $h_{2}={\hat{A}}^{2}XW_{1}=\left[\begin{array}{cc} -1 & 0\\ 2 & 1 \end{array}\right]$, then $\text{Pmax}\left(h_{1},h_{2}\right)=\left[\begin{array}{cc} 1 & 0\\ 2 & 1 \end{array}\right]$.

In the information dissemination and fusion process, both the first-order neighborhood features and the high-order neighborhood features are fully considered. Therefore, the classification accuracy should be improved.

### 3.2. Our Proposed Model

**Figure 2:**
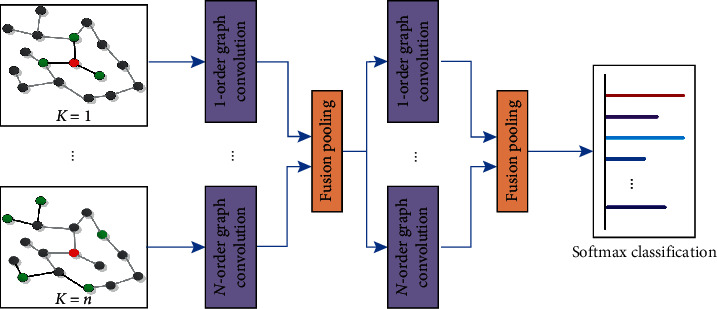
HLHG mode. The graph convolutional network layer of the HLHG model consists of two convolutional layers and information fusion pooling. The input parameters are from the first-order to the *n*-th order neighborhoods. When *n* = 1, the model degenerates into a classical graph convolution GCN model. When the neighborhood order is *n* = 2, it is called the HLHG-2 model, and its input parameters are the 1st order neighborhood and the 2nd order neighborhood. When the neighborhood order is *n* = 3, it is called the HLHG-3 model, and its input parameters are the 1st order neighborhood, the 2nd order neighborhood, and the 3rd order neighborhood.

In Figure 2, we propose the high-order graph convolutional network model to fuse the high-order messages that pass through the graph network. The model consists of an input layer, two graph convolutional layers, and an information fusion pooling layer that is connected to the graph convolutional layer. The softmax function is used for the multiclassification output.

The proposed model extends the classical GCN model [6] to the graph neural network of higher-order neighborhoods. Each node in the model can get its representation from its neighborhood and integrate messages. The system model is as follows:

(4) $$\begin{array}{c} Y=ℱ\left(Pm\left(\hat{A}\sigma \left(H^{\left(l+1\right)}\right)W_{l+1},\cdots ,{\hat{A}}^{\left(p\right)}\sigma \left(H^{\left(l+1\right)}\right)W_{l+1}\right)\right), \end{array}$$

where *p* is the order of the neighborhoods, ${\hat{A}}^{\left(p\right)}$ = ${\hat{A}}^{\left(p-1\right)}\hat{A}$, *σ*(.) is the activation function, function ℱ(.) denotes the softmax function. Parameter *W*_*l*+1_ is the trainable weight parameter of layer (*l* + 1) in the graph network, and function *Pm*(.) represents Pmax(.), which denotes the hybrid high-order and low-order of the information fusion. When parameter *l* is equal to 0, H1=PmaxA^H0W0, ⋯,A^pH0W0, which is the output of the first convolutional layer of the graph propagation model. In addition, *H*^(0)^ = *X* ∈ *R*^*n*×*r*^, which represents the initial input of our model.

In the preliminary experiment, we found that the two-layer high- and low-order mixed graph convolution is better than the one-level high- and low-order mixed graph convolution, and stacking more layers does not significantly improve the accuracy of the graph recognition task. Therefore, this paper uses a 2-layer graph convolution layer. In further experiments, we validate *p*=2 and *p*=3 in equation (4) for our HLHG models. In the supervised learning and unsupervised learning classification tasks, our HLHG models show very good performance and achieve a good balance between the classification accuracy and computational complexity. We also validate that at *p*=4 and *p* > 4, the classification accuracy is not significantly improved. Therefore, we only analyze and implement our model for *p*=2 and *p*=3 in the following sections.

In equation (4), the model with *p*=2, that is, the hybrid model of the 1st and 2nd order neighborhoods, is called the HLHG-2 model. The model with *p*=3, that is, the hybrid model of the 1st, 2nd, and 3rd order neighborhoods, is called the HLHG-3 model.

In the HLHG-2 model, it assumes that the graph convolutional network has 2 convolutional layers and the activation function is Relu. Then, the output Y of the HLHG-2 model can be expressed as follows:

(5) $$\begin{array}{c} Y=ℱ\left[Pm\left[\hat{A}\left(\text{Relu}\left(M2\right)\right)W_{2},{\hat{A}}^{2}\left(\text{Relu}\left(M2\right)\right)W_{2}\right]\right], \end{array}$$

where $M2=P\max\left(\hat{A}XW_{1},\;{\hat{A}}^{2}XW_{1}\right)$ and *Pm* denotes the fusion pooling *P*max.

The same as with the HLHG-2 model, the output Y of the HLHG-3 model can be expressed as follows:

(6) $$\begin{array}{c} Y=ℱ\left[Pm\left[\hat{A}T,{\hat{A}}^{2}T,{\hat{A}}^{3}T\right]\right], \end{array}$$

where *𝒯*=(Relu(*M*3))*W*_2_ and $M3=P\max\left(\hat{A}XW_{1},\;{\hat{A}}^{2}XW_{1},{\hat{A}}^{3}XW_{1}\right)$.

For a large-scale graph network, it is unacceptable to directly calculate ${\hat{A}}^{3}={\hat{A}}^{\left(2\right)}\hat{A}=\hat{A}\hat{A}\hat{A}$. Therefore, we calculate ${\hat{A}}^{3}XW_{1}=\hat{A}\left(\hat{A}|\left(\hat{A}|X\right)\right)$ *W*_1_. In general, the dimension of $\hat{A}X$ is less than $\hat{A}$, and this procedure avoids large-scale matrix multiplication operations.

Therefore, our HLHG model has a 2-layer graph network, and the iterative expression of the 2nd order neighborhood is as follows:

(7) $$\begin{array}{c} Y=\text{softmax}\left(\hat{A}\text{Relu}\left(H\right)W_{2},{\hat{A}}^{2}\text{Relu}\left(H\right)W_{2}\right), \end{array}$$

where $H=P\max\left(\hat{A}XW_{1},\;{\hat{A}}^{2}XW_{1}\right)$. We use *P*max as our fusion pooling operator, which assumes the maximum value in the corresponding element.  Algorithm 1 shows how to fuse the different order neighbors.

We use the multiclassified cross entropy as the loss function of our HLHG model, $L=-\sum_{i}{\tilde{y}}_{i}\log\left(q_{i}\right)$, where $\tilde{Y}$ is the labeled samples. The graph neural network trainable weights *W*_1_ and *W*_2_ are trained using gradient descent. In each training iteration, we perform the batch gradient descent.

### 3.3. Computational Complexity and Parameter Quantity

In the large-scale graph network, the adjacency matrix is $\hat{A}\in R^{n\times n}$. It is difficult to directly calculate ${\hat{A}}^{\left(p\right)}$. To reduce the computational complexity, we iteratively calculate ${\hat{A}}^{\left(p\right)}$. For higher orders, the right to left iterative multiplication procedure is ${\hat{A}}^{\left(p\right)}H^{\left(l\right)}W_{l}=\left({\hat{A}}^{\left(p\right)}H^{\left(l\right)}\right)W_{l}=\hat{A}\left({\hat{A}}^{\left(p-1\right)}H^{\left(l\right)}\right)W_{l}$. For example, when *p*=1, ${\hat{A}}^{\left(1\right)}H^{\left(0\right)}=\hat{A}X\in R^{n\times r}$. When *p*=2, ${\hat{A}}^{\left(2\right)}H^{\left(1\right)}=\hat{A}\left(\hat{A}|X\right)\in R^{n\times r}$.

In the proposed model, the input feature of the graph network is *X* ∈ *R*^*n*×*r*^. The weight of the first convolutional layer is *W*_1_ ∈ *R*^*r*×*r*_1_^, and the weight of the second layer is *W*_2_ ∈ *R*^*r*_1_×*r*_2_^. Then, the input of the first convolutional layer is *H*^(0)^=*X* ∈ *R*^*n*×*r*^ where the parameter *r* represents the dimension of the input feature. For example, *r*_1_ denotes the number of hidden neurons in the first convolutional layer and *r*_2_ denotes the number of hidden neurons in the second convolutional layer. In our HLHG model, the trainable weight parameters are shared in the same convolutional layer. Therefore, in the first convolutional layer, the output dimension after the convolutional operator is the same. That is, $\hat{A}XW_{1}\in R^{n\times r_{1}}$, ${\hat{A}}^{\left(2\right)}XW_{1}\in R^{n\times r_{1}}$, and ${\hat{A}}^{\left(k\right)}XW_{1}\in R^{n\times r_{1}}$, where *k* is the order of the adjacency matrix $\hat{A}$.

In the *l*-th convolutional layer, ${\hat{A}}^{\left(k\right)}H^{\left(l\right)}W_{l}\in R^{n\times r_{l}}$, where *r*_*l*_ denotes the number of hidden neurons in the *l*-th convolutional layer. It assumes that $\hat{A}$ is a sparse matrix with *m* nonzero elements. For the *l*-th convolutional layer of our HLHG, the computational complexity is *O*(*r*_*l*_ × *k* × *m* × *r*_*l*−1_) and the quantity of trainable weight is *O*(*r*_*l*_ × *r*_*l*−1_).

The total computational complexity of our HLHG model is *O*(∑_*l*_^*j*^(*r*_*l*_ × *k* × *m* × *r*_*l*−1_)), and the total number of trainable parameters is *O*(∑_*l*_^*j*^(*r*_*l*_ × *r*_*l*−1_)), where parameter *j* denotes the total number of convolutional layers and *l* denotes the *l*-th convolutional layer. When *l*=1, *r*_0_ represents the feature dimensions of the datasets and *r*_*l*_ represents the number of hidden neurons in the *l*-th convolutional layer. For all the datasets, *r*_0_ ≫  *r*_*l*_; therefore, we only consider the first convolutional layer when we compare the computational complexity and number of parameters.

Compared to [14], we set fewer filters to maintain a similar computational complexity and the number of parameters is less via weight sharing for both the lower-order and higher-order convolutions.

## 4. Experiments

We conduct experiments in order to verify that our HLHG model can be applied to supervised learning and semisupervised learning. On the text network datasets, we compare our model with the state-of-the-art methods using supervised learning. On the citation network datasets, we compare our model with the state-of-the-art methods using semisupervised learning. For all experiments, we construct a 2-layer graph convolutional network of our model using TensorFlow. The code and data are available on GitHub.

### 4.1. Supervised Text Network Classification

We conduct supervised learning on five benchmark text graph datasets to compare the classification accuracy of HLHG with the graph convolutional neural network and other deep learning approaches.

#### 4.1.1. Datasets

In our supervised experiments, the 20-Newsgroups (20NG), Ohsumed, R52 and R8 of Reuters 21578, and Movie Review (MR) are used to verify the proposed models. These datasets are publicly available on the web and are widely used as test-verified datasets. The summary statistic features of the text network are shown in Table 1.

These benchmark text datasets were processed by Yao et al. [21], who converted the text datasets into graph network structures. Then, they used preprocessing to construct the adjacency matrix of the graph network input and input parameters. The dataset is divided into a training dataset and a test dataset in the same way.

#### 4.1.2. Baselines and Experimental Setting

We compare our HLHG with the following approaches: the convolutional neural network with pretrained vectors (CNN-rand) [22], the LSTM model with pretrained vectors (LSTM-pre) [23], the predictive text embedding for text classification (PTE) [24], the fast text classifier (fastText) [25], the simple word embedding model with simple pooling strategies (SWEM) [26], the label-embedding attentive model for text classification (LEAM) [27], the graph CNN model with the Chebyshev filter (GCN-C) [13], the graph CNN model with the spline filter (GCN-S) [5], the graph CNN model with the Fourier filter (GCN-F) [11], and the graph convolutional network for text classification (text GCN) [21]. The baseline models were tested by Yao et al. [21].

In our HLHG-2 model, we set the dropout rate = 0.2. The learning rate is updated from Adam [28] during the training process. In our model, we set the L2 loss weight as 0, and we adopt early stopping. We set the learning rate to 0.02 for the R8 dataset, and the learning rates of the remaining datasets are all set to 0.01. We set different epochs for different datasets. The number of epochs in the R52 dataset is 350. The number of epochs in the OH and 20NG datasets is 200, and the number in the R8 and MR datasets is 60. In the HLHG-2 model, we set the number of hidden neurons in the 1st convolutional layer as 128 for all datasets.

Except for the parameters in Table 2, the other parameters are the same as in the HLHG-2 model.

For our HLHG-3, we set the number of hidden neurons in the first convolutional layer to 128 except for the MR dataset, which is set to 64. To obtain better training results, we separately set different hyperparameters such as the dropout rate, learning rate, and number of epochs for different datasets (see Table 2). In addition, the other parameters of HLHG-3 are the same as those in HLHG-2.

We construct the graph network for our HLHG-2 and HLHG-3 models, and the feature matrix and other parameters are the same as those by Yao et al. [21].

#### 4.1.3. Results

We show supervised text classification accuracies for the five datasets in Table 3. We demonstrate how our model performs on common splits that were taken from Yao et al.'s study [21].

Table 3 presents the classification accuracies and standard deviations of our models and the benchmark on the text network data. In general, our HLHG-2 and HLHG-3 achieve high levels of performance. Specifically, they achieve the best performances on R52, OH, 20NG, and R8. Compared to the best performing approach, the proposed models yield worse accuracies on the MR dataset. In general, the HLHG-3 and HLHG-2 models perform equally well. More specifically, the 3rd order HLHG has slightly better classification accuracy than the 2nd order HLHG on most datasets. However, the performance difference is not very large. Overall, the proposed architecture with hybrid high- and low-order neighborhoods has good classification performance, which indicates that it effectively preserves the topological information of the graph, and it also obtains a high-quality representation of the nodes.

The benchmark test results are copied from [8]. The mean standard deviation of our model is the average of 100 runs.

Table 4 shows the comparison of the network complexity and the number of parameters with the Text GCN [21]. Our HLHG can match the Text GCN with respect to computational complexity while requiring fewer parameters than the Text GCN. As described in Section 3.3, the number of features in the dataset is much larger than the number of neurons in the hidden convolutional layer. Therefore, we only compare the computational complexity and number of parameters of the first convolutional layer in our HLHG model. In Table 4, Comp. and Params represent the computational complexity and the number of parameters in the first layer of the graph convolutional network, respectively. In the computational complexity results, the first constant denotes the number of neurons in the first convolutional layer and the second constant denotes the order of the adjacency matrix. The parameter *m* denotes the number of nonzero entries of the sparse regularization adjacency matrix. The parameter *r* denotes the feature dimension of the nodes in the graph network.

In the Text GCN [21], the number of hidden neurons in the first convolutional layer is 200; therefore, the complexity and params are 200. In our HLHG-2 model, 128 denotes the number of hidden neurons in the first convolutional layer and 2 represents the highest order of HLHG-2. In our HLHG-3 model, 128 and 64 denote the number of hidden neurons in the first convolutional layer and 3 represents the highest order of the corresponding model. The result in Table 4 shows that our HLHG-3 model has better computational complexity for the MR dataset. Because of the weight sharing in the different order neighborhoods, our HLHG models require fewer trainable weight parameters. Especially on the MR dataset, the number of parameters is only 1/3 of that of the Text GCN [21].

### 4.2. Semisupervised Node Classification

We conduct semisupervised learning on three benchmark citation network datasets to compare the node classification accuracy of HLHG with some classical approaches and with some graph convolutional neural network approaches. The graph semisupervised learning corresponds to the process of “label” spreading on citation networks.

#### 4.2.1. Datasets

In semisupervised node classification, we use the CiteSeer, Cora, and PubMed citation network datasets [29]. In these citation datasets, the nodes represent the articles that were published in the corresponding journal. The edges between the two nodes represent references from one article to another, and the tags represent the topics of the articles. The citation link constructs an adjacency matrix. Those datasets have low label rates. The summary statistic features of the citation graph are shown in Table 5.

#### 4.2.2. Baselines and Experimental Setting

We compare our HLHG with the same baseline methods as by Abu-El-Haija et al. [15] and Yang et al. [30]. The baselines are as follows: manifold regularization (ManiReg) [31], semisupervised embedding (SemiEmb) [32], label propagation (LP) [33], skip-gram-based graph embeddings (DeepWalk) [34], the iterative classification algorithm (ICA) [35], Planetoid [30], HO [14], and MixHop [15].

For the HLHG-2 model, we use the following parameters for the citation datasets (Cora, CiteSeer, and PubMed): 16 (number of hidden units), 0.5 (dropout rate), 0.0005 (L2 regularization), 10 (early stopping), 300 (number of epochs), and 0.01 (learning rate).

For tthe HLHG-3 model, we set different numbers of hidden neurons for the different datasets. We set 8 hidden neurons for the CiteSeer dataset to reduce the computational complexity and the number of parameters, and set 10 hidden neurons for the Cora and PubMed datasets to capture richer features. The hyperparameters of the HLHG-3 are set as shown in Table 6.

#### 4.2.3. Results

In the semisupervised experiments, we train and test our models on those citation network datasets following the methodology that was proposed by Yang et al. [30]. The classification accuracy is the average of 100 runs with random weight initializations.

The benchmark test results were copied from [15, 30]. The mean standard deviation of our model is the average of 100 runs.

In Table 7, the node classification accuracies that are above the line are copied from Abu-El-Haija [14, 15] and Yang et al. [30]. The values below the line are our HLHG models.  ± represents the standard deviation of 100 runs with different random initializations. These splits utilize only 20 labeled nodes per class during training. We achieve the best test accuracies of 82.7% and 71.5% on the Cora and CiteSeer datasets, respectively. Compared with other high-order graph convolutional neural networks [14, 15] on the same datasets, they get the high-order information using linear combinations of features from farther distances. Our HLHG model acts nonlinearly to get the high-order neighborhood information.

In Table 8, we compare the network complexity and the number of parameters with the other high-order graph convolutional networks and the classic GCN. The result shows that our model has the same computational complexity as other approaches. With respect to the number of parameters, our HLHG-3 model has fewer parameters than the GCN [6]. The reason is that our model shares the weights in the same layer among the different order neighborhood matrixes.

## 5. Conclusion

In this paper, we propose a hybrid lower-order and higher-order GCN model for the supervised classification of text network datasets and for semisupervised classification in a citation network. In our model, we propose a novel nonlinear information fusion layer to combine the low- and higher-order neighborhoods. To reduce the number of parameters, we propose sharing the weights in the same convolutional layer with different order neighborhoods. Experiments on the two network datasets suggest that HLHG has the capability to fuse higher-order neighborhoods for supervised classification and semisupervised classification. Our model significantly outperforms the benchmarks. We also find that the computational complexity and the number of parameters are less than those of the high-order method. In order to obtain more neighborhood information, we could use more higher-order adjacency matrix. However, the direct use of higher orders may lead to oversmoothing problems. Therefore, in future research work, we will extend our HLHG models to fuse graph attention networks [36] to develop a deeper graph convolutional network.

## Figures and Tables

**Algorithm 1:**
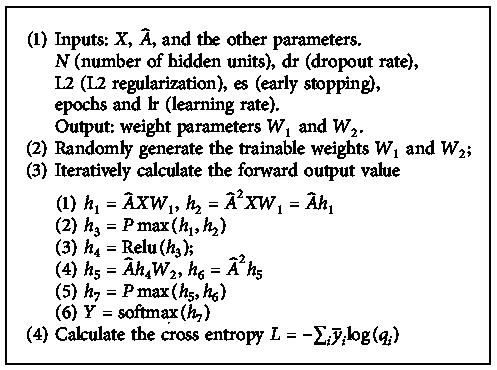
Iterative calculation for HLHG-2.

**Table 1:** Text network datasets.

Datasets	*C*	*D*	Tr	Te	*N*
R52	52	9,100	6,532	2,568	17,992
OH	23	7,400	3,357	4,043	21,557
20NG	20	18,846	11,314	7,532	61,603
R8	8	7,674	5,485	2,189	15,362
MR	2	10,662	7,108	3,554	29,426

*C* indicates the category, *D* is the total number of texts, Tr is the training set, Te is the test set, and *N* is the number of vertices of the graph network.

**Table 2:** The hyperparameters in our HLHG-3 model.

Datasets	Dropout	Learning rate	Epochs
R52	0.6	0.005	950
OH	0.2	0.01	230
20NG	0.0	0.01	210
R8	0.2	0.005	300
MR	0.1	0.01	80

**Table 3:** Text network classification accuracy.

Methods	R52	OH	20NG	R8	MR
CNN-rand [22]	87.59	58.44	82.15	95.71	**77.75**
LSTM [23]	85.54	41.13	65.71	93.68	75.06
LSTM-pre [23]	90.48	51.10	75.43	96.09	77.33
PTE [24]	90.71	53.58	76.74	96.69	70.23
fastText [25]	92.81	57.70	79.38	96.13	75.14
SWEM [26]	92.94	63.12	85.16	95.32	76.65
LEAM [27]	91.84	58.58	81.91	93.31	76.95
GCN-C [13]	92.75	63.86	81.42	96.99	77.22
GCN-S [5]	92.74	62.82	—	96.80	76.99
GCN-F [11]	93.20	63.04	—	96.89	76.74
Text GCN [21]	93.56	68.36	86.34	97.07	76.74
HLHG-2 (ours)	94.21 ± 0.14	69.16 ± 0.19	**86.57** **±** **0.08**	**97.25** **±** **0.10**	75.95 ± 0.14
HLHG-3 (ours)	**94.33** **±** **0.16**	**69.36** **±** **0.24**	86.35 ± 0.24	97.25 ± 0.12	76.49 ± 0.32

**Table 4:** Comparison of network computational complexity and the number of parameters.

Approaches	Comp.	Params
Text GCN [21]	O (200 × 1 × *m* × *r*)	O (200 × *r*)
HLHG-2 (ours)	O (128 × 2 × *m* × *r*)	O (128 × *r*)
HLHG-3 (ours)	O (64 × 3 × *m* × *r*) (MR dataset)	O (64 × *r*) (MR dataset)
	O (128 × 3 × *m*×*r*) (other datasets)	O (128 × *r*) (other datasets)

**Table 5:** Citation network datasets.

Datasets	*N*	*E*	*F*	*L*	*C*
Cora	2708	5429	1433	0.052	7
CiteSeer	3327	4732	3703	0.036	6
PubMed	19717	44338	500	0.003	3

*N* means the number of nodes of citations, *E* means the number of edges between citations, *F* means the number of features of the nodes, *L* denotes the labeling rate, and *C* denotes the number of classes.

**Table 6:** The hyperparameters of HLHG-3.

Datasets	Dropout	Learning rate	Early stopping	Epochs
Cora	0.5	0.01	No	500
CiteSeer	0.5	0.005	5	500
PubMed	0.6	0.01	1	200

**Table 7:** Citation network classification test accuracy.

Approaches	Cora	CiteSeer	PubMed
ManiReg [31]	59.5	60.1	70.7
SemiEmb [32]	59.0	59.6	71.1
LP [33]	68.0	45.3	63.0
DeepWalk [34]	67.2	43.2	65.3
ICA [35]	75.1	69.1	73.9
Planetoid [30]	75.7	64.7	77.2
GCN [6]	81.5	70.3	79.0
HO-3 [14]	81.6 ± 0.47	71.2 ± 0.94	80.0 ± 0.64
HO-4 [14]	81.6 ± 0.63	71.2 ± 0.84	80.1 ± 0.65
MixHop [15]	81.8 ± 0.62	71.4 ± 0.81	80.0 ± 1.10
MixHop (learned) [15]	81.9 ± 0.40	71.4 ± 0.81	*80.8* ± *0.58*
HLHG-2 (ours)	*82.7* ± *0.28*	*71.5* ± *0.22*	79.1 ± 0.18
HLHG-3 (ours)	82.7 ± 0.29	71.5 ± 0.39	79.3 ± 0.15

**Table 8:** Comparison of network complexity and number of parameters.

Methods	Comp.	Params
GCN [6]	*O* (16 × *m* × *r*)	*O* (16 × *r*)
HO-3 [14]	*O* (10 × 3 × *m* × *r*)	*O* (10 × 3 × *r*)
HO-4 [14]	*O* (10 × 4 × *m* × *r*)	*O* (10 × 4 × *r*r)
MixHop [15]	*O* (20 × 2 × *m*×r)	*O* (20 × 3 × *r*)
MixHop (learned) [15]	*O* (20 × 2 × *m* × *r*)	*O* (60 × *r*)
HLHG-2 (ours)	*O* (16 × 2 × *m* × *r*)	*O* (16 × *r*)
HLHG-3 (ours)	*O* (8 × 3 × *m* × *r*) (CiteSeer)	*O* (8 × *r*) (CiteSeer)
	*O* (10 × 3 × *m* × *r*) (Cora, PubMed)	*O* (10 × *r*) (Cora, PubMed)

## Data Availability

The Supervised Text Network Classification data used to support the findings of this study have been deposited in the repository DOI:10.1609/aaai.v33i01.33017370. The Semisupervised Node Classification data used to support the findings of this study have been deposited in the repository DOI:10.1609/aimag.v29i3.2157
